# Altered Metabolic Profiles Associate with Toxicity in SOD1^G93A^ Astrocyte-Neuron Co-Cultures

**DOI:** 10.1038/s41598-017-00072-4

**Published:** 2017-03-03

**Authors:** Gabriel N. Valbuena, Massimo Tortarolo, Caterina Bendotti, Lavinia Cantoni, Hector C. Keun

**Affiliations:** 1 Department of Surgery and Cancer, Faculty of Medicine, Imperial College London, Du Cane Road, London, W12 0NN UK; 2 Department of Neuroscience, IRCCS- Istituto di Ricerche Farmacologiche “Mario Negri”, 20156 Milan, Italy; 3 Department of Molecular Biochemistry and Pharmacology, IRCCS- Istituto di Ricerche Farmacologiche “Mario Negri”, 20156 Milan, Italy

## Abstract

Non-cell autonomous processes involving astrocytes have been shown to contribute to motor neuron degeneration in amyotrophic lateral sclerosis. Mutant superoxide dismutase 1 (SOD1^G93A^) expression in astrocytes is selectively toxic to motor neurons in co-culture, even when mutant protein is expressed only in astrocytes and not in neurons. To examine metabolic changes in astrocyte-spinal neuron co-cultures, we carried out metabolomic analysis by ^1^H NMR spectroscopy of media from astrocyte-spinal neuron co-cultures and astrocyte-only cultures. We observed increased glucose uptake with SOD1^G93A^ expression in all co-cultures, but while co-cultures with only SOD1^G93A^ neurons had lower extracellular lactate, those with only SOD1^G93A^ astrocytes exhibited the reverse. Reduced branched-chain amino acid uptake and increased accumulation of 3-methyl-2-oxovalerate were observed in co-culture with only SOD1^G93A^ neurons while glutamate was reduced in all co-cultures expressing SOD1^G93A^. The shifts in these coupled processes suggest a potential block in glutamate processing that may impact motor neuron survival. We also observed metabolic alterations which may relate to oxidative stress responses. Overall, the different metabolite changes observed with the two SOD1^G93A^ cell types highlight the role of the astrocyte-motor neuron interaction in the resulting metabolic phenotype, requiring further examination of altered met abolic pathways and their impact on motor neuron survival.

## Introduction

Amyotrophic lateral sclerosis (ALS) is a neurodegenerative disease characterized by the loss of motor neurons, leading to progressive neuromuscular impairment and death, usually within 2–3 years after diagnosis. Around 10% of ALS cases are due to inherited genetic mutations, with those in the Cu-Zn superoxide dismutase gene (SOD1) first to be associated with the disease. Rodents expressing SOD1 mutations invariably develop a motor syndrome with pathological and symptomatic features of the human disease, therefore representing the best experimental models of ALS available. To date, the process by which mutant SOD1 leads to toxicity has not yet been fully resolved, although oxidative stress, excitotoxicity, dysregulation of energy metabolism, and mitochondrial dysfunction have been extensively studied as potential pathogenic mechanisms, based on studies of patient tissues and experimental models of the disease^1–3^. A complex multifactorial pathogenesis would be compatible with a key toxic role for global derangements in metabolism.

Metabolomic and proteomic analysis of biofluids from ALS patients have revealed altered profiles and are being applied in search of biomarkers for diagnosis^4–6^. However, there is still limited information available on the effect of mutant SOD1 expression on metabolism in the different cell types of the nervous system. Our group has previously shown that while expression of wild type human SOD1 in the NSC-34 cell line reinforced metabolic responses to stress, this process was dysregulated with expression of mutant SOD1^G93A^ and was coupled to loss of cell viability, supporting a role for metabolic impairments in motor neuronal dysfunction^7^. The NSC-34 line and primary motor neurons share similar metabolic pathways^8^, but the degree of functional relevance of results in proliferating cell lines compared to primary cell cultures may vary. Furthermore, single culture of neurons cannot reproduce the cooperative metabolic processes occurring between different cell types that is fundamental to brain homeostasis.

In the nervous system, neurons and the surrounding astrocytes differ markedly in their metabolic functions^9^ and are organized as a functional unit profoundly influencing each other’s gene expression^10^. The highly complex metabolic cross-talk between these two cell types is vital for neuronal health and synaptic plasticity^11^. These include the synthesis of glutathione, the main defence against oxidative stress^12^, the glutamate-glutamine cycle, through which astrocytes control cerebral glutamate concentrations, and the exchange of substrates such as products of branched chain amino acid (BCAA) metabolism and lactate^13^ to fuel neuronal metabolism^14^ and glutamate synthesis^15, 16^.

Several studies, mainly based on mutant SOD1 experimental models, have indicated that ALS is a non-cell-autonomous disease^17, 18^. Astrocytes, in particular, are likely to take part in processes leading to motor neuron injury and contribute to disease progression^19–22^. We have previously shown that motor neurons isolated from mutant SOD1^G93A^ embryos do not die when spinal neurons are cultured on their own, but become selectively vulnerable if co-cultured with transgenic SOD1^G93A^ astrocytes^23^.

In this study, we aimed to determine whether the expression of mutant SOD1 protein altered metabolic interactions between astrocytes and spinal neurons and to examine how this associates with the progressive loss of motor neuron viability. We used co-cultures of astrocytes and spinal neurons obtained from SOD1^G93A^ embryos and their non-transgenic counterparts, a model that we have previously characterized to exhibit spontaneous and selective loss of motor neurons^23^. We measured the change in extracellular metabolite concentrations compared to fresh media of co-cultures of non-transgenic and transgenic SOD1^G93A^ astrocytes and spinal neurons, providing information on net uptake or net release of metabolites over the periods of incubation. Samples were taken at two different timepoints, corresponding to varying degrees of motor neuron loss. We show that metabolite profiles in astrocyte-neuron co-cultures were significantly modified when mutant SOD1^G93A^ was expressed, which may relate to the loss of viability to which motor neurons appear uniquely susceptible.

## Results

### Co-cultures expressing SOD1^G93A^ either in the astrocytes, in the neurons, or in both exhibit selective motor neuron death 6 days after neuron plating

**Figure 1:**
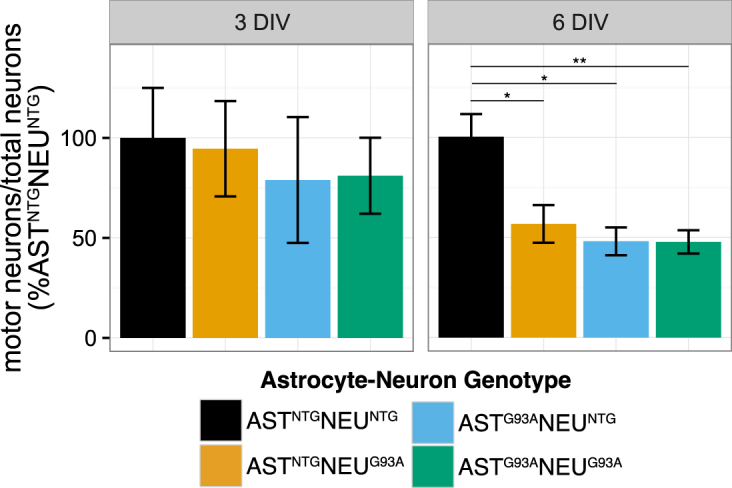
Selective motor neuron death in co-cultures with SOD1^G93A^ expression after 6 days *in vitro*. Co-cultures of astrocytes and spinal neurons were grown for 3 days *in vitro* (3 DIV) or 6 days (6 DIV) after neuron seeding. SOD1^G93A^ expression in the neurons (AST^NTG^NEU^G93A^), in astrocytes (AST^G93A^NEU^NTG^), or in both cell types (AST^G93A^NEU^G93A^) leads to significant selective motor neuron death compared to fully non-transgenic co-culture (AST^NTG^NEU^NTG^) after 6 DIV. Figure shows the ratio of motor neurons to total spinal neurons expressed as a percentage of the respective AST^NTG^NEU^NTG^ co-culture (presented as mean ± SEM, n = 4–6, *p < 0.05, **p < 0.01 after a Student’s t-test).

We previously reported that SOD1^G93A^ expression in both astrocytes and motor neurons determines selective motor neuron death under co-culture conditions^23^. In agreement with previous findings, there were significantly fewer large motor neurons in the AST^G93A^NEU^G93A^ compared to the AST^NTG^NEU^NTG^ co-cultures grown for six days *in vitro* (DIV) after neuron plating, while the number of total spinal neurons remained unchanged (Fig. 1). Genotype-mixed co-cultures, where SOD1^G93A^ was expressed only in the astrocytes (AST^G93A^NEU^NTG^) or only in the neurons (AST^NTG^NEU^G93A^) also had significant motor neuron death at 6 DIV similar to fully transgenic co-cultures (AST^G93A^NEU^G93A^). No statistically significant selective motor neuron death was observed at 3 DIV.

### Multivariate analysis reveals varying metabolic patterns between co-cultures at the two timepoints (3 DIV and 6 DIV)

Metabolic profiles of media from co-culture were generated using ^1^H NMR spectroscopy. While it is not possible to assert a specific change in uptake or release from any given cell type in co-culture from such an analysis, it is possible to define net change in consumption or release of metabolites in the co-culture as a whole and to relate this to the genotypes of the cells in co-culture.

**Figure 2:**
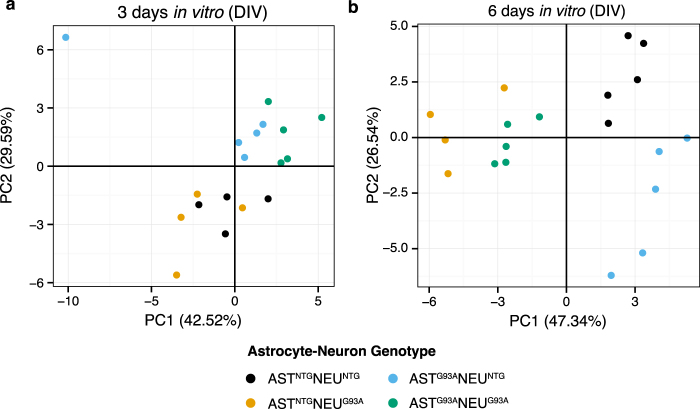
Co-culture metabolomes show clustering based on astrocyte genotype after 3 DIV and clustering based on neuron genotype after 6 DIV. PCA scores plot for metabolomes of astrocyte-spinal neuron co-cultures (n = 4–5) (**a**) after 3 DIV, and (**b**) after 6 DIV (n = 4–5). The percentage of variance explained by each component is shown in parentheses.

The metabolite net consumption and release profiles in the co-cultures were analysed by principal components analysis, and the influence of astrocyte genotype, neuron genotype, and an interaction between the two factors on the first two principal components was assessed by a two-way ANOVA. At 3 DIV, a significant interaction effect was found in PC1 (21.9% of total variation, F(1,14) = 4.98, p = 0.042). PC2, on the other hand, exhibited a significant effect for astrocyte genotype (67.16% of total variation, F(1,14) = 30.42, p = 7.60E-05), seen in the scores plot as a separation between co-cultures with NTG astrocytes in the negative PC2 space and those with SOD^G93A^ astrocytes in the positive PC2 space (Fig. 2a). Analysis of the loadings showed that PC2 scores were most influenced by 3-hydroxyisobutyrate, citrate, and lactate (high positive PC2 loadings), as well as by the BCAAs isoleucine, leucine, valine, and choline, and 3-methyl-2-oxovalerate on the negative axis (Supplementary Fig. 1A).

At 6 DIV (Fig. 2b), a significant effect on PC1 from neuron genotype was observed, representing 89.6% of total variation (F(1,15) = 197.92, p = 4.78E-10). A smaller effect on PC1 from astrocyte genotype was also found to be significant, representing 6.0% of total variation (F(1,15) = 7.89, p = 0.013). PC2 scores were found to be significantly influenced by astrocyte genotype (34.8% of total variation, F(1,15) = 14.53, p = 0.0017) as well as by an interaction effect (22.4% of variation, F(1,15) = 8.57, p = 0.0104). The loadings show that PC1 scores are driven most strongly by phenylalanine, lactate, glucose, methionine, and choline (Supplementary Fig. 1B), and PC2 scores are driven most by valine, citrate, formate, and 3-hydroxyisobutyrate.

The separation patterns from PCA are also consistent with results from a two-way ANOVA of individual metabolite measurements, where calculation of the percentage of total variation attributable to either the astrocyte genotype, neuron genotype, or an interaction between the two shows that differences between the co-cultures at 3 DIV were influenced more strongly by astrocyte genotype (Supplementary Fig. 2). At 6 DIV, on the other hand, more of the variation in metabolite concentrations were driven by neuron genotype.

### SOD1^G93A^ expression leads to increased glucose consumption in astrocytes and neurons and altered media concentrations of lactate

Changes in metabolite concentrations were then considered individually to identify any changes potentially associated with motor neuron death in the co-culture model. The fully transgenic AST^G93A^NEU^G93A^ co-culture is the most comparable to the conditions in nervous tissue in the SOD1^G93A^ mouse model and to SOD1 fALS patients, as in these cases mutant SOD1 protein is expressed in both astrocytes and neurons. The metabolite changes were also compared with those in culture media incubated with either NTG or mutant SOD1^G93A^ astrocytes in single culture. Any differences with astrocytes in single culture in metabolite accumulation or depletion in the medium were expected to be due to the presence of neurons in the co-cultures or due to metabolic interactions between two different cell types^24^.

**Figure 3:**
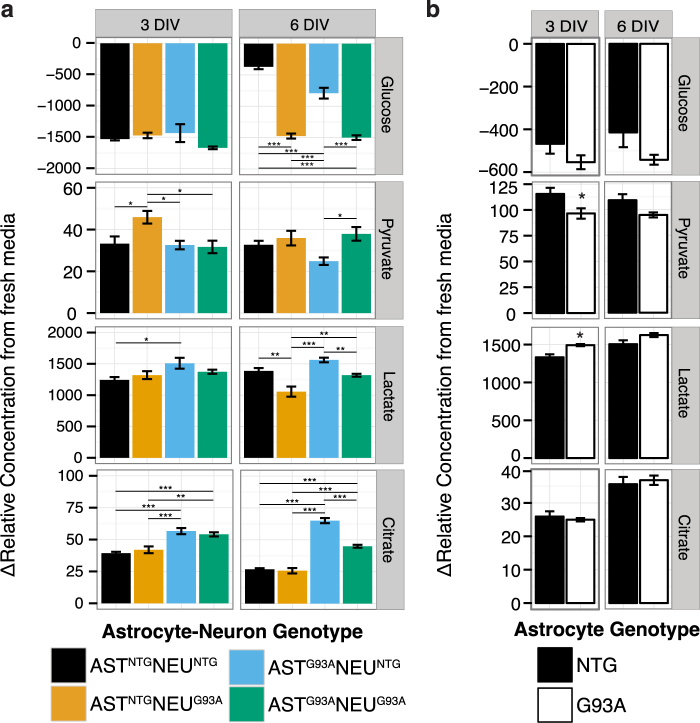
The extracellular metabolome reveals shifts in concentrations of energy metabolites in co-cultures with SOD1^G93A^ neurons. Change in metabolite concentrations in the medium of (**a**) astrocyte-spinal neuron co-cultures and (**b**) astrocyte single cultures. Asterisks denote statistically significant differences (**a**) between co-culture genotypes after a two-way ANOVA followed by a Tukey HSD post hoc test or (**b**) between astrocyte genotypes after a Student’s t-test (*p < 0.05, **p < 0.01, ***p < 0.001). All figures show mean ± s.e.m. (n = 4–5).

No significant differences were observed in net glucose uptake in the co-cultures at 3 DIV (Fig. 3a), although extracellular net lactate production was higher in co-cultures with SOD1^G93A^ astrocytes (27.2% of total variation influenced by astrocyte genotype, F(1,15) = 6.45, p = 0.0236 after a two-way ANOVA). For astrocytes in single culture at the same timepoint, media lactate accumulation was significantly elevated in the SOD1^G93A^ compared to NTG cells, while pyruvate was significantly decreased (Fig. 3b).

At 6 DIV, we observed significantly higher net glucose uptake in all co-cultures where one or both cell types expressed SOD1^G93A^ compared to AST^NTG^NEU^NTG^ (Fig. 3a). Glucose depletion was also significantly increased in co-cultures with SOD1^G93A^ neurons compared to their NTG counterparts (AST^NTG^NEU^G93A^ vs AST^NTG^NEU^NTG^ and AST^G93A^NEU^G93A^ vs AST^G93A^NEU^NTG^). However, despite a large difference in net glucose uptake between the fully-NTG co-culture AST^NTG^NEU^NTG^ and the fully-transgenic co-culture AST^G93A^NEU^G93A^, there was no significant difference in lactate accumulation between the two.

The two-way ANOVA indicates that lactate metabolism at 6 DIV were influenced by both astrocyte genotype (25.7%% of total variation, p = 7.27E-4) and neuron genotype (51.5% of total variation, p = 2.5E-5). We observed increased media lactate accumulation in co-cultures with SOD1^G93A^ astrocytes compared to NTG, with significantly higher net lactate accumulation in the AST^G93A^NEU^G93A^ co-cultures compared to AST^NTG^NEU^G93A^ (p < 0.01, Fig. 3a). We also saw lower lactate accumulation in co-cultures with SOD1^G93A^ neurons compared to NTG, with significantly lower lactate accumulation in AST^NTG^NEU^G93A^ co-cultures compared to AST^NTG^NEU^NTG^ (p < 0.01), as well in the AST^NTG^NEU^G93A^ and AST^G93A^NEU^G93A^ compared to AST^G93A^NEU^NTG^ (p < 0.001 and p < 0.01 respectively). Interestingly, lactate accumulation was highest in the AST^G93A^NEU^NTG^ co-cultures even though the increase in net glucose uptake compared to the fully non-transgenic co-culture AST^NTG^NEU^NTG^ was much smaller than that observed in co-cultures with SOD1^G93A^ neurons (AST^NTG^NEU^G93A^ and AST^G93A^NEU^G93A^, p < 0.001). Net pyruvate release was also significantly lower in the AST^G93A^NEU^NTG^ compared to AST^G93A^NEU^G93A^ co-cultures. Taken together, these observations suggest increased glucose demand with SOD1^G93A^ expression in either astrocytes or neurons in the co-culture system, and decreased net lactate accumulation when SOD1^G93A^ neurons are present.

Extracellular citrate accumulation was significantly increased in co-cultures with SOD1^G93A^ astrocytes (AST^G93A^NEU^NTG^ and AST^G93A^NEU^G93A^) at both timepoints (Fig. 3a), with astrocyte genotype influencing 76.5% of total variation in citrate at both 3 DIV (F(1,14) = 50.8, p = 5.11E-6) and at 6 DIV (F(1,15) = 325.05, p = 1.41E-11). The link between citrate release and astrocyte genotype is consistent with highly active synthesis of citrate from pyruvate carboxylation^25^ and high rates of citrate release^26^ previously observed in astrocytes, as well as the fact that citrate is neither released by neurons nor used as a neuronal substrate^27^. However, this effect with SOD1^G93A^ expression is triggered by interactions in co-culture since there was no significant difference in citrate release when the astrocytes were grown in single culture (Fig. 3b).

We also observed reduced net release of phenylalanine, serine, and asparagine at 6 DIV (Supplementary Fig. 3), with an effect associated with neuron genotype (51–69% of total variation, p < 0.001), and a small effect associated with astrocyte genotype only for asparagine (11.6% of total variation, p < 0.05). The release of these amino acids were reduced in co-cultures with SOD1^G93A^ neurons compared to their NTG counterparts. We found no significant differences in concentrations of these metabolites when astrocytes were in single culture, and in the co-cultures at 3 DIV. As these changes occur alongside increased glucose uptake with SOD1^G93A^ neurons, it is possible that these metabolites are being used in anaplerotic reactions to facilitate continued flux through the TCA cycle by supplying intermediates.

Overall, the results in co-culture indicate that SOD1^G93A^ increases the demand for fuel substrates whether it is expressed in astrocytes or neurons. SOD1^G93A^ expression in astrocytes alone leads to increased glycolytic activity (with increased glucose uptake and lactate release). In co-culture, we also observe a more marked increase in glucose uptake in the presence of SOD1^G93A^ expressing neurons, although this is associated with lower net lactate release. Possible reasons for the lower net lactate release include increased lactate utilization, as neurons are known to take up lactate for use as an energy substrate. Glucose may also be used to increase flux through the pentose phosphate pathway (PPP), consistent with the limited ability of neurons to increase glycolysis^28^. These complex rearrangements of cellular bioenergetics appear to coincide with the selective loss of motor neuron viability in co-cultures when SOD1^G93A^ is expressed in either or both cell types.

### Branched-chain amino acid metabolism is perturbed differently in astrocytes and neurons by SOD1^G93A^ expression

The BCAAs leucine, isoleucine, and valine are extensively metabolized in the brain and spinal cord. They are first deaminated by branched-chain aminotransferases (BCAT) to branched-chain keto-acids (BCKAs) in a reversible reaction, transferring the α-amino group to α-ketoglutarate (forming glutamate). The BCKAs are then converted to branched chain acyl-CoAs by the branched-chain α-ketoacid dehydrogenase complex (BCKDC) in a reaction requiring NAD^+^. Glutamate is converted back into α-ketoglutarate by glutamate dehydrogenase (GDH), also consuming NAD^+^ in the process. Cultured primary astroglial cells from newborn rodent brains primarily express the mitochondrial isozyme of BCAT (BCATm), while in neuronal cells, only the cytosolic isozyme is found (BCATc). In contrast, BCKCD is expressed in the mitochondria of both cell types^29, 30^.

**Figure 4:**
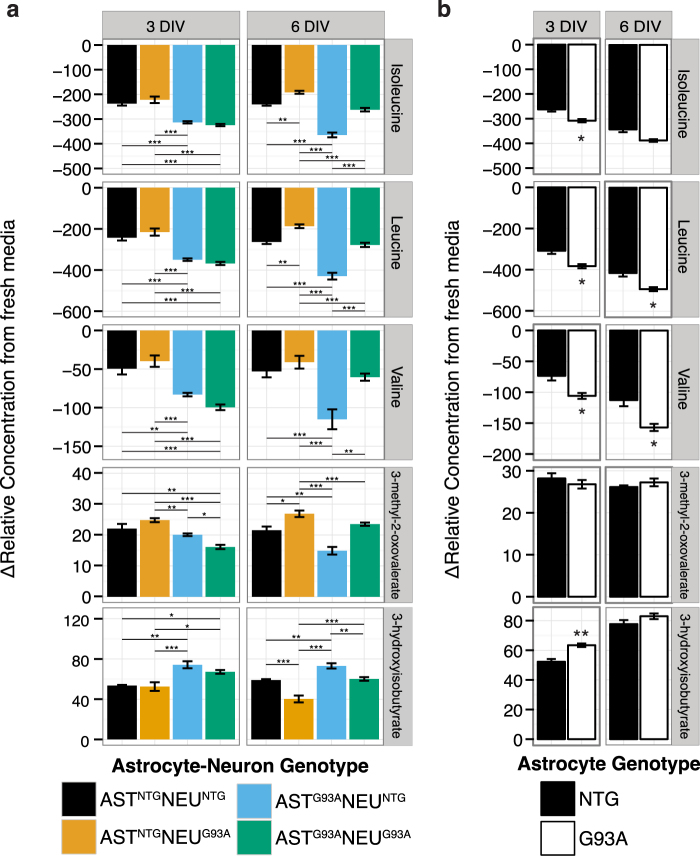
Reduced net uptake of branched chain amino acids in co-cultures with SOD1^G93A^ neurons is accompanied by increased net release of 3-methyl-2-oxovalerate. Concentrations of BCAAs, the branched chain keto acid product of isoleucine, 3-methyl-2-oxovalerate, and the downstream valine metabolite 3-hydroxyisobutyrate in the medium of (**a**) astrocyte-spinal neuron co-cultures and (**b**) astrocyte single cultures. Asterisks denote statistically significant differences (**a**) between co-culture genotypes after a two-way ANOVA followed by a Tukey HSD post hoc test or (**b**) between astrocyte genotypes after a Student’s t-test (*p < 0.05, **p < 0.01, ***p < 0.001). All figures show mean ± s.e.m. (n = 4–5).

In the astrocyte single cultures, BCAA net uptake was elevated in SOD1^G93A^ astrocytes vs. NTG astrocytes at both 3 DIV (p < 0.01) and 6 DIV (p < 0.01 except for isoleucine, Fig. 4b). There was also higher net release of 3-hydroxyisobutyrate (a valine metabolite downstream of BCKA processing) in SOD1^G93A^ astrocyte cultures compared to NTG at 3 DIV (p < 0.01, Fig. 3b). In the co-cultures, a large percentage of total variation in BCAA uptake at 3 DIV was found to be from astrocyte genotype (78–89%, p < 0.001 after a two-way ANOVA). At this timepoint, we observed significantly higher net uptake of leucine, isoleucine, and valine in co-cultures with SOD1^G93A^ astrocytes (Fig. 4a). This was accompanied by significantly lower net release in the co-cultures with SOD1^G93A^ astrocytes of 3-methyl-2-oxovalerate, the α-keto acid intermediate in isoleucine degradation, suggesting increased full oxidation of isoleucine. Differences in levels of 3-hydroxyisobutyrate at 3 DIV were also largely influenced by astrocyte genotype (67.3% of total variation, F(1,14) = 35.6, p = 3.47E-5), with higher 3-hydroxyisobutyrate accumulation in co-cultures with SOD1^G93A^ astrocytes (Fig. 4a).

However, the variation in BCAA uptake (as well as 3-methyl-2-oxovalerate release) at 6 DIV was significantly influenced not only by astrocyte genotype (representing 36–55% of total variation, p < 0.001 after a two-way ANOVA), but also by neuron genotype (representing 26–41% of total variation, p < 0.01) and to a much lesser extent, an interaction between the two (4–10% of total variation, p < 0.05, Fig. 4a). While the co-cultures with SOD1^G93A^ astrocytes continued to have higher BCAA consumption compared to their NTG counterparts, we observed decreased BCAA net uptake when comparing co-cultures with SOD1^G93A^ neurons with their NTG counterparts. This was accompanied by significantly increased 3-methyl-2-oxovalerate net release in the AST^NTG^NEU^G93A^ co-cultures vs. AST^NTG^NEU^NTG^, indicating a potential block in BCKA processing in co-cultures with SOD1^G93A^ neurons. We also continued to see significantly higher 3-hydroxyisobutyrate accumulation in co-cultures with SOD1^G93A^ astrocytes compared to their counterparts with NTG astrocytes (AST^G93A^NEU^NTG^ vs AST^NTG^NEU^NTG^ and AST^G93A^NEU^G93A^ vs AST^NTG^NEU^G93A^) representing 43.5% of total variation (F(1,15 = 50.25, p = 3.69E-6). However, we also observed an effect from neuron genotype at this timepoint, accounting for 42.0% of total variation (F(1,15 = 48.64, p = 4.47E-6). We observed significantly lower 3-hydroxyisobutyrate in co-cultures with SOD1^G93A^ neurons compared to their NTG counterparts (AST^NTG^NEU^G93A^ vs. AST^NTG^NEU^NTG^, p < 0.001 and AST^G93A^NEU^G93A^ vs. AST^G93A^NEU^NTG^ p < 0.01). This would also be consistent with a block in BCKA processing, as that would lead to accumulation of BCKAs and reduced levels of downstream metabolic products such as 3-hydroxyisobutyrate.

A possible cause of these phenomena could be increased utilization of BCAAs in SOD1^G93A^ astrocytes, which would contribute towards enriching the pool of acetyl-CoA and succinyl-CoA entering the TCA cycle. This would be consistent with the upregulation of glycolysis and lactate release in SOD1^G93A^ astrocytes described in the previous section, with BCAA catabolism supporting continued TCA cycle flux through anaplerosis. The transfer of nitrogen from BCAAs to glutamate and further on to glutamine has been observed in cultured astrocytes^31^. Expression of BCATm, GDH and BCKDC in astrocyte mitochondria may permit their interaction as an organized supramolecular complex or metabolon^32, 33^. This would promote not only BCKA oxidation, but also the cycling of nitrogen through glutamate (which only occurs when BCKA products are oxidized) and formation of ammonia.

Studies with leucine on rodent astrocyte and neuron co-cultures identify a metabolic cycle involving the exchange of BCAAs and BCKAs linking the two cell types^16, 34^. This operates in parallel with the glutamate-glutamine cycle, allowing amino nitrogen that exits astroglia as glutamine to be replenished by amino nitrogen from BCAAs released by neurons. This exchange depends on BCAT activity in both cell types. Glutamate formed by BCATm in astrocytes may be used to form glutamine, which when released is then taken up by neurons to regenerate glutamate. In the neurons, the BCAA transaminase reaction favors the re-amination of the BCKAs, consuming glutamate in the process^16^. The regenerated BCAA is then released by the neuron for uptake in the astrocytes, where it is used to produce glutamate through BCATm, completing the cycle.

Our results suggest potential perturbations in this metabolic cycle due to SOD1^G93A^ expression in the co-cultures, and over time in culture. In the transgenic co-cultures, the decrease in BCAA uptake and metabolism coincided with increased motor neuron death, suggesting that a block in BCAA regeneration and/or of metabolism of BCKA may contribute to toxicity.

### The glutamine-glutamate cycle is perturbed in co-cultures expressing SOD1^G93A^

**Figure 5:**
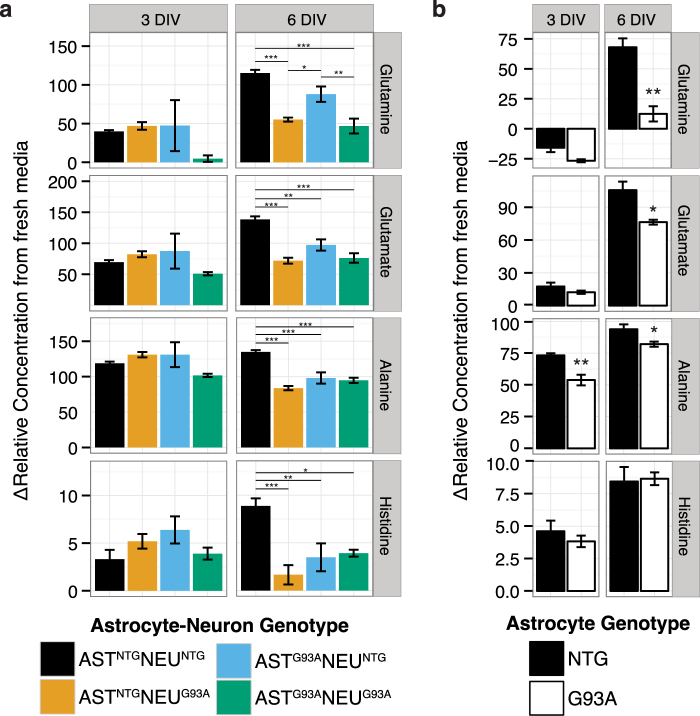
Glutamate and glutamine metabolism is perturbed by SOD1^G93A^ expression. Concentrations of glutamine and glutamate, as well as alanine and histidine in the medium of (**a**) astrocyte-spinal neuron co-cultures and (**b**) astrocyte single cultures. Asterisks denote statistically significant differences (**a**) between co-culture genotypes after a two-way ANOVA followed by a Tukey HSD post hoc test or (**b**) between astrocyte genotypes after a Student’s t-test (*p < 0.05, **p < 0.01, ***p < 0.001). All figures show mean ± s.e.m. (n = 4–5).

When astrocytes were in single culture, we observed significantly lower glutamate concentrations in the medium of SOD1^G93A^ astrocytes compared to NTG (Fig. 5b). While astrocytes are known to strongly take up glutamate, this is an energy-consuming process that can be impaired during energy failure^35^. As such, the possibility that the decrease in glutamate is due to reduced release by SOD1^G93A^ astrocytes cannot be excluded entirely. We also observed higher net uptake of glutamine at 3 DIV and lower net release at 6 DIV in SOD1^G93A^ astrocytes compared to NTG, which was not expected, given that increased BCAA uptake (as described in the previous section) has been reported to increase glutamine release^34^. A decrease in glutamine synthetase activity is unlikely, as lower concentrations of extracellular glutamate (as seen in the SOD1^G93A^ astrocytes) should correspond to increased activity of glutamine synthetase^27^, and any inhibition of glutamine synthetase should increase extracellular alanine concentrations^36^, an effect we did not observe. These results suggest that SOD1^G93A^ expression in the astrocytes may alter the homeostasis of glutamine and glutamate pools. Maintenance of this homeostasis involves several enzymes abundant in astrocytes^35^ namely glutamine synthetase and glutaminase (for the synthesis and catabolism of glutamine), the transaminases (alanine aminotransferase, ALT and aspartate aminotransferase, AAT, forming glutamate from α-ketoglutarate) and GDH (oxidizing glutamate to α-ketoglutarate and producing NADH).

Glutamine and glutamate homeostasis in the co-cultures is more complex, as these metabolites are also exchanged between astrocytes and neurons according to the glutamate-glutamine cycle. This is a non-stoichiometric process coupling neurotransmission to metabolism, and is thus crucial for the health of neuronal cells. Briefly, the glutamate released by neurons is taken up by astrocytes, where it has a range of possible metabolic fates^35^, including conversion to glutamine by glutamine synthetase (expressed exclusively in astrocytes) or as a substrate for energy production or amino acid synthesis. Glutamine is then readily released by astroglial cells and taken up by neurons, where glutaminase converts it back to glutamate for neurotransmission or metabolism to support the neuronal TCA cycle.

In the co-cultures, we observed no significant differences in media glutamine or glutamate accumulation at 3 DIV, although glutamine concentrations in the medium in the fully-transgenic AST^G93A^NEU^G93A^ co-cultures were markedly lower than the other co-cultures (Fig. 5a). At 6 DIV, we observed significant differences in glutamine accumulation, with neuron genotype accounting for 63.6% of total variation, F(1,15) = 42.06, p = 1.03E-5, and astrocyte genotype contributing to 11.4% of total variation, F(1,15) = 7.53, p = 0.015. Glutamate accumulation was also significantly altered, with 50.5% of total variation influenced by neuron genotype (F(1,15) = 35.85, p = 2.49E-5), 13.9% by astrocyte genotype (F(1,15) = 13.85, p = 0.007), and 14.5% by their interaction (F(1,15) = 10.3, p = 0.006). Net glutamine and glutamate release were significantly lower in both co-cultures with SOD1^G93A^ neurons compared to the fully-NTG AST^NTG^NEU^NTG^ co-culture. Glutamine and glutamate concentrations were comparable in both co-cultures with SOD1^G93A^ neurons, suggesting that SOD1^G93A^ in this cell type alone is sufficient to reduce media accumulation of these metabolites. It is also of note that the decrease in glutamine and glutamate accumulation was smaller in the AST^G93A^NEU^NTG^ co-culture (which exhibited a marked increase in BCAA uptake) compared to the co-cultures with SOD1^G93A^ neurons, supporting a possible link between BCAA uptake and concentrations of glutamine and glutamate.

Alanine homeostasis is also affected by glutamate concentrations, as its production through transamination by ALT of pyruvate requires glutamate, either formed by other transamination reactions in the cytosol or through reductive amination of α-ketoglutarate by GDH in the mitochondria. The reduced net alanine release we observe suggests that the flow of glutamate from these pathways may be reduced with SOD1^G93A^ expression. Alanine release at both 3 and 6 DIV was significantly lower in SOD1^G93A^ astrocytes in single culture compared to NTG (Fig. 5b). The alanine release:glucose uptake ratio was also lower in SOD1^G93A^ astrocytes single culture compared to NTG, suggesting a potential preference for alanine utilization rather than release in SOD1^G93A^ astrocytes (Supplementary Fig. 4). Interestingly, at 6 DIV there were significantly lower alanine concentrations in all co-cultures with SOD1^G93A^ expression compared to the fully-non transgenic co-culture AST^NTG^NEU^NTG^ (p < 0.001). The two-way ANOVA indicates that alanine release in the co-cultures was strongly influenced by neuron genotype (35.7% of total variation, F(1,14) = 26.29, p = 1.24E-4), an interaction between astrocyte and neuron genotype (30.5% of total variation, F(1,14) = 22.46, p = 2.64E-4), and to a much lesser extent, astrocyte genotype (13.4% of total variation, F(1,14) = 9.83, p = 0.0068, Fig. 5a). This suggests that SOD1^G93A^ expression may increase the demand for alanine and limit its release into the medium in both cell types. The efficient production of alanine and its export by neurons constitutes a complementary way to transfer nitrogen from neurons to astrocytes for glutamine synthesis, playing a role in the glutamate-glutamine cycle^24^. The decreased alanine accumulation in the medium of SOD1^G93A^ expressing co-cultures may also favor derangements in this cycle.

Interestingly, we also observed a significant and substantial decrease in histidine accumulation in all SOD1^G93A^ expressing co-cultures compared to the fully-NTG AST^NTG^NEU^NTG^ co-culture (Fig. 5a). This large decrease in histidine was apparently triggered by co-culture, as there were no differences observed in astrocytes in single culture (Fig. 5b). The oxidative catabolism of histidine leads to the formation of glutamate, making it another potential contributor to glutamate production. Together, the metabolite reductions observed in the medium of SOD1^G93A^ expressing co-cultures support the presence of alterations to glutamine and glutamate metabolism, modifying the glutamate-glutamine cycle; this phenotype coincides with the marked decrease in SOD1^G93A^ motor neuron viability.

### Altered concentrations of methionine and choline in the media of co-cultures with SOD1^G93A^ neurons

**Figure 6:**
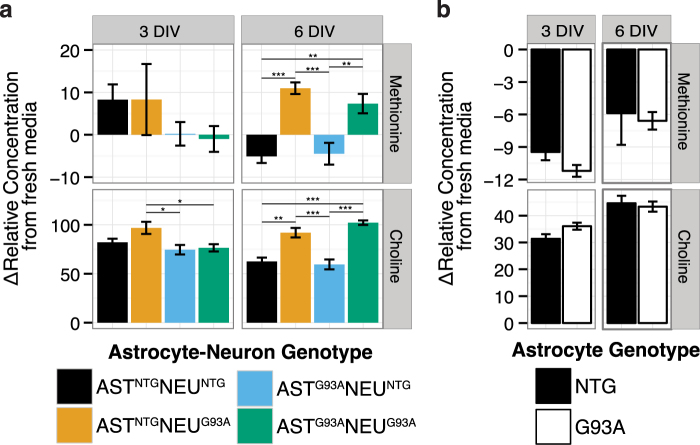
Increased net release of methionine and choline in co-cultures with SOD1^G93A^ neurons. Concentrations of methionine and choline in the medium of (**a**) astrocyte-spinal neuron co-cultures and (**b**) astrocyte single cultures. Asterisks denote statistically significant differences (**a**) between co-culture genotypes after a two-way ANOVA followed by a Tukey HSD post hoc test or (**b**) between astrocyte genotypes after a Student’s t-test (*p < 0.05, **p < 0.01, ***p < 0.001). Figures show mean ± s.e.m. (n = 4–5).

When we examined concentrations of methionine, an essential amino acid, and choline, which among its many metabolic functions is a precursor of betaine (involved in homocysteine remethylation to methionine), we found no significant differences in the astrocytes in single culture (Fig. 6b). In the co-cultures, however, we found that those with NTG neurons exhibited net uptake of methionine while the co-cultures with SOD1^G93A^ neurons had net methionine release (Fig. 6a). This difference was statistically significant after a two-way ANOVA, with neuron genotype contributing to 73.1% of total variation (F(1,15) = 43.87, p = 8.1E-6). Methionine catabolism generates cysteine, a process that is upregulated in oxidative stress conditions to sustain glutathione synthesis. The net release of methionine in the co-cultures with SOD1^G93A^ neurons supports the possibility that catabolism of sulfur-containing amino acids is impaired in SOD1^G93A^ neurons.

We also observed increased media choline in co-cultures with SOD1^G93A^ neurons at 6 DIV, with neuron genotype accounting for 80.2% of total variation (F(1,15) = 77.64, p = 2.57E-7). Interestingly, we also observed increased net choline release in the AST^NTG^NEU^G93A^ co-culture compared to both co-cultures with SOD1^G93A^ astrocytes (p < 0.05), making it one of the few metabolites to have altered concentrations at the earlier timepoint and potentially of interest as an early marker of metabolic alteration.

## Discussion

The co-culture approach has been instrumental in establishing a role for non-cell autonomous effects in the pathogenic process of ALS^18^. Our study presents a new perspective on this interaction, revealing that SOD1^G93A^ expression may affect the balance of reciprocal metabolic interactions in astrocytes and neurons in co-culture. We examined extracellular metabolite levels in various combinations of non-transgenic and SOD1^G93A^ astrocytes and neurons, and observed marked changes in metabolites involved in energy generation, the glutamate-glutamine cycle, and redox homeostasis, all processes fundamental for maintaining neuronal activity^28^.

The presence of mutant SOD1 in either the astrocytes or the neurons led to alterations in concentrations of metabolites involved in energy generation. We observed higher glucose net uptake in co-cultures with SOD1^G93A^ astrocytes at 6 DIV. Astrocytes are constitutively glycolytic, providing neurons with energy substrates and GSH precursors in addition to their role in neurotransmitter clearance and the provision of glutamine for neurotransmitter synthesis. Under conditions of energy and oxidative stress, astrocytes adapt by upregulating glucose metabolism^37, 38^, allowing for increased use of glucose either to maintain ATP levels or in the PPP to increase reducing power for antioxidant synthesis. We found no clear difference in lactate release with SOD1^G93A^ astrocytes in single culture consistent with findings by Ferraiuolo, *et al.* ^3^. They found decreased capacity for lactate secretion in SOD1^G93A^ astrocytes when separated from neurons after long-term (14 day) co-culture, however we do not have comparable lactate measurements as we have not examined changes in metabolite concentrations in the medium of astrocytes separately after co-culture. Our measurements represent combined contributions from the two cell types during co-culture, with the differences in net release representing the balance of metabolite uptake and release between the cell types in co-culture. At 6 DIV, we observed higher net lactate release associated with SOD1^G93A^ astrocytes in the co-cultures, although we cannot determine from these experiments alone whether this is due to changes in the astrocytes alone, whether it is due to complementary changes in neuronal lactate processing in the presence of SOD1^G93A^ astrocytes, or whether it is part of a longer-term process of alteration in both cell types that extends to 14 days and beyond. These highlight the complexity of cellular interactions in this model, where the interactions themselves may induce processes shifting metabolism in each cell type that would not be observed when the different cell types are grown separately.

In addition, the shifts in media glucose concentrations we observed at 6 DIV suggest that glucose homeostasis in the co-culture environment may be much more complex than a continuous consumption process over time. Glucose consumption in the co-cultures with SOD1^G93A^ neurons was comparable at 6 DIV to that in the equivalent co-cultures at 3 DIV, with no substantial additional net glucose consumption at 6 DIV. Media concentrations of glucose were also higher in co- cultures with NTG astrocytes at 6 DIV than in co-cultures at 3 DIV. As the cultures have been kept in the same media over their respective incubation periods, the increased media glucose concentrations at 6 DIV does not indicate reduced uptake from an intermediate refresh. The absence of a fresh source of glucose would suggest that by 6 DIV, glucose was being released into the media when astrocytes were cultured with non-transgenic neurons, and that this process did not occur when the astrocytes are cultured with SOD1^G93A^ neurons instead. Under that scenario, the additional glucose may come from glycogen, which in the CNS is predominantly found in the astrocytes^39^. Astrocytic glycogen is mobilized in cases where the neuronal energy demand exceeds supply^40^. However, the initial glucose concentration was 33 mM, and glucose concentrations continued to be above expected *in vivo* concentrations during the period of culture. There was no complete depletion of glucose in the medium at 6 DIV; as such, the changes in media glucose uptake are not due to limits on substrate availability or hypoglycemia. As there is limited information on the effects of continuous astrocyte-neuron co-culture on metabolism, let alone its evolution over time or the responses to pathogenic ALS mutations, extensive further characterization of this model will be required to understand the nature of these changes to glucose homeostasis.

The shifts in BCAA uptake and glutamine/glutamate generation, on the other hand. suggest a block in the mechanisms involved in processing both sets of amino acids, as it has been identified that BCAT, BCKDC, and GDH interact as an organized supramolecular complex or metabolon^32^ in the mitochondria at least in the astrocytes. The reduced glutamine and glutamate release in co-culture is consistent with reduced glutamate generation from BCAT, which would limit the glutamate available to regenerate glutamine via glutamine synthetase. This link is crucial, as BCAAs provide 30–50% of the nitrogen for glutamate synthesis in astrocytes *in vivo* ^41^. The BCKA accumulation from a block in BCKCD may trigger reductions in BCAT activity to limit cellular BCKA concentrations, as branched-chain ketoaciduria is known to lead to severe neurotoxicity in maple syrup urine disease, with evidence that impaired energy metabolism and oxidative stress play a role in pathogenesis^42^. The block in BCKDC may be due to limits on the NAD^+^ pool available, with GDH competing favorably for reducing power and decoupling the BCAT-GDH interaction from BCKDC. Alternatively, the BCKDC or BCAT may be directly affected. A regulatory motif sensitive to changes in the redox environment is present in BCAT^43^.

The coupling of BCAA and glutamate metabolism is consistent with earlier observations that BCAAs can act as allosteric activators of GDH *in vitro* ^44, 45^. BCAA supplementation has been trialed as a treatment for ALS to activate glutamate conversion by GDH and limiting excitotoxicity. A small pilot trial demonstrated significant improvements in maintenance of extremity muscle strength and continued ability to walk^46^. Later trials, however, have led to either no impact on survival^47, 48^, with Tandan *et al*. reporting a higher rate of loss of pulmonary function with BCAA treatment, or to increased mortality with BCAA treatment^49^. Adverse outcomes with BCAA supplementation in patients would be consistent with our observations as increasing BCAA concentrations in a system with impaired BCAA metabolism would lead to accumulation of BCAAs and BCKAs to toxic levels, particularly as plasma BCAA concentrations have been observed to increase to 500–1377% after dosing^50^. Further work is needed to examine whether impairments in these biochemical interactions contribute to the toxicity observed in ALS.

Citrate was shown to be elevated in co-cultures containing astrocytes, which synthesize citrate *de novo* at levels 20-fold above that of neurons^26^. Citrate accumulation in the media may indicate impairment of aconitase activity, as observed in fluorocitrate neurotoxicity^51^. Aconitase levels may be reduced in ALS, as the aconitase protein was enriched in the insoluble fraction of protein extracts (representing the insoluble protein aggregates) from spinal cords of SOD1^G93A^ mice, as well as of ALS patients^52^.

Glutamate has been proposed to mediate motor neuron death in ALS through excitotoxicity^53–55^, but while these results suggest a key potential block in the glutamate processing capacity of cells in ALS, media glutamate and glutamine concentrations highlight potential incompatibilities with classical excitotoxicity theory. Extracellular glutamate concentrations are expected to be increased in excitotoxicity, but we observe reduced extracellular glutamate in the co-cultures containing SOD1^G93A^ cells as well as in SOD1^G93A^ astrocytes in single culture compared to their non-transgenic counterparts. However, glutamate concentrations may not be the sole determinant of vulnerability to excitotoxicity, as extracellular glutamate was not increased in a co-culture model showing motor neuron loss similar to ours^20^ and increased glutamate concentrations have not consistently been demonstrated in CSF^54, 56, 57^. Further work will be necessary to establish the extent of any excitotoxic effects or vulnerabilities in this model, and any role of perturbations to the coupled BCAA and glutamate processing pathways observed here in the excitotoxicity process. Reference 58 has been left in the text erroneously after revisions made in the process of paper review. Please remove reference 58 from the manuscript text and references.

The other key metabolic changes may also be associated with the response to oxidative stress, which has been consistently observed in experimental models of SOD1 ALS and is implicated in ALS pathogenesis^58–60^. The increased 3-methyl-2-oxovalerate we observed in co-cultures with SOD1^G93A^ neurons suggest that a redox imbalance may shift the directionality of BCKCD in reducing/oxidizing the branched-chain keto acids^61^. This metabolic response to oxidative stress is consistent with previous observations in astrocyte-motor neuron co-cultures, where the toxicity caused by mutant SOD1 astrocytes was partially due to increased ROS and RNS production^62, 63^ and prevented by treatments to increase antioxidant defenses. In NSC-34 cells expressing SOD1^G93A^, lower glutathione levels were associated with altered energy metabolism and decreased glutamate content^64^. Motor neurons have high metabolic demands and increased ROS formation^59^, and their structural and metabolic specialization makes them particularly vulnerable to neurotoxicity from stressors like the introduction of high levels of oxidative stress (whether through intrinsic generation or triggered by external stimuli) and impairments to energy metabolism, whether triggered by SOD1^G93A^ expression or from external stimuli. The inability to control these stressors may then lead to feed-forward cycles with the other proposed mechanisms, triggering the further generation of oxidative stress and perpetuating the cycle of toxic insults that ultimately lead to motor neuron death.

While we identify net changes in extracellular metabolite levels associated with the presence or absence of a particular SOD1 genotype in astrocytes and/or neurons in this study, it is limited by the fact that the measurements represent combined contributions from the two cell types during co-culture, and as such, these metabolic alterations cannot be conclusively determined to be due to changes in uptake or release in one or both cell types from these measurements alone. This study is also limited by the high non-physiological concentrations of glucose in the media, however, this has been optimized for astrocyte and neuron survival *in vitro* and is comparable to previous studies. We also note that Ara-C was used in these experiments to inhibit the proliferation of contaminating microglia. Ara-C was used uniformly across the different experimental conditions in this study, and as such, any influence on the metabolic profile is expected to be exerted similarly in these experimental models. Ara-C primarily affects DNA synthesis, a pathway we do not explore in this study, although in the future, it may be of interest to examine any specific metabolic impacts of Ara-C.

Overall, the co-culture approach has given clearer answers when probing binary endpoints like cell death and the initiation of apoptosis, but the effects on cellular metabolism in each cell type are much more complex. Further examination of basal metabolite consumption and release when astrocytes and neurons are cultured together, as well as metabolic changes in each cell type during co-culture under conditions comparable to *in vivo* nutrient availability will be necessary to fully characterize the changes in the metabolic processes we identify in this study and their relationship to motor neuron toxicity in astrocyte-neuron co-cultures.

## Conclusion

**Figure 7:**
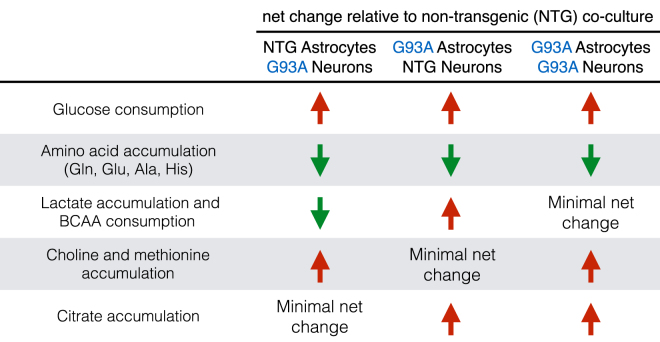
Altered metabolic processes in astrocyte- neuron co-cultures with SOD1^G93A^ expression. Summary figure presents metabolic changes in co-cultures with SOD1G93A expression only in the spinal neurons, only in astrocytes, and in both astrocytes and spinal neurons compared to the fully non-transgenic co-culture.

Overall, different metabolic adaptations between the astrocytes and neurons in co-culture were observed in the presence of SOD1^G93A^ both in each cell type individually and when SOD1^G93A^ was expressed in both cell types (Fig. 7). The results indicate the presence of stress on energy metabolism, as well as alterations to the glutamine-glutamate cycle and to redox homeostasis. The co-culture approach reveals changes stimulated by the presence of a mixture of cell types, which cannot easily be studied by looking at astrocytes and neurons separately. However, the added complexity of the mixed cultures also makes specific interpretation of observations more challenging. Further characterization of metabolic changes relating to the interaction between the two cell types in the context of mutant SOD1, will be required to understand the role of metabolism in the selective motor neuron toxicity that occurs in mixed culture. Despite these limitations, this work underscored the role that astrocytes may play in the development of neurotoxicity in ALS. Improved understanding of the nature and impact of these processes in astrocytes may open opportunities for the development of protective interventions aimed at improving the ability of astrocytes to maintain neuroprotective metabolic pathways.

## Materials and Methods

### Animal model

Procedures involving animals and their care were conducted in accordance with institutional guidelines of the Mario Negri Institute for Pharmacological Research, Milan, Italy (IRFMN), in compliance with national (D.L. no. 116, G.U. suppl. 40, Feb. 18, 1992, Circular No. 8, G.U., 14 luglio 1994) and international laws and policies (EEC Council Directive 86/609, OJ L 358, 1 Dec. 12, 1987; NIH Guide for the Care and use of Laboratory Animals, U.S. National Research Council, 1996). All experimental protocols proposed were reviewed and approved by the IRFMN Animal Care and Use Committee (IACUC). The mice were bred and maintained in a specific-pathogen-free environment. We used NTG and SOD1^G93A^ mice originally purchased from Jackson Laboratories (B6SJL-Tg-SOD1-G93A-1Gur) and maintained on a C57BL/6JOlaHsd (Harlan) genetic background at the IRFMN^23^. The genotype of the embryos was determined by PCR analysis of DNA extracted from tissue biopsies.

### Co-culture model

Astrocyte-spinal neuron co-cultures were prepared as previously described^65^.

#### Astrocyte culture preparation

E13-E14 NTG and SOD1^G93A^ embryos were used*.* As we have previously demonstrated that SOD1^G93A^ cortical- or spinal cord-derived astrocytes have comparable effects on the viability of SOD1^G93A^ motor neurons in the co-culture model^23^, we used cortical astrocytes in this study.

Cortices were dissected from the brain and the meninges removed. They were then mechanically dissociated using a fire-polished Pasteur pipette in 2 ml of Hank’s balanced salt solution (HBSS) containing 33 mM glucose. Residual meninges and debris were eliminated by a 10 min-sedimentation. The supernatant was then centrifuged at 1000 rpm for 10 min and the resulting pellet was resuspended in astrocyte culture medium (DMEM/F12 containing 2 mM L-glutamine, 33 mM glucose, 5 μg/ml gentamycin, and 10% heat-inactivated horse serum). Cells were seeded (125,000/cm^2^) into 48-well plates coated with 1.5 μg/ml poly-L-ornithine and pre-conditioned with complete culture medium. Astrocytes were grown at 37 °C in humidified atmosphere with 5% CO_2_ until they reached confluence (2–3 weeks). Proliferation was then stopped by 10 μM cytosine arabinoside (Ara-C) treatment. Repeated washes with HBSS during the growth, 12 h-orbital shaking at 200 rpm and 10 μM Ara-C treatment rendered astrocyte cultures virtually free of microglia and oligodentrocytes, as demonstrated by immunocytochemical analysis with specific markers (Cd11b and oligodendrocyte marker 04 respectively, Tortarolo, *et al.* ^55^). The resulting cultures are thus highly-enriched astroglial cultures^66^. These cultures have been well-characterized in previous work. They have been shown to express GFAP marker, glutamate transporters, and do not present activation signs such as the release of inflammatory mediators^23, 55^

#### Spinal neuron and co-culture preparation

Cortical astrocyte-spinal neuron co-cultures were prepared as previously described^65^. Spinal cords of E13-E14 embryos were dissected and mechanically dissociated in HBSS with 33 mM glucose. The cells were centrifuged onto a 4% BSA cushion at 1000 rpm for 10 min and the pellet resuspended in co-culture medium: Neurobasal medium (Gibco) with 2 mM L-glutamine, 33 mM glucose, 5 μg/ml gentamycin, 1 ng/ml BDNF, 25 μg/ml insulin, 10 μg/ml putrescine, 30 nM sodium selenite, 2 μM progesterone, 100 μg/ml apo-transferrin, 10% heat-inactivated horse serum, and 10 μM Ara-C. Cells were seeded (250,000 cells/cm^2^) into 48-well plates onto the pre-established astrocyte confluent layer to obtain spinal neuron-cortical astrocyte co-cultures.

Motor neurons obtained from E13-14 embryos are fully differentiated and express the specific transcription factors Hb9 and Islet-1/2. Few days after plating, they show adult characteristics such as profuse dendrite and axon outgrowth^20, 67, 68^. An average of 1.2% of mixed spinal neurons were SMI32-positive motor neurons with large cell bodies (maximum diameter > 20 µm), extensive dendritic arborisation, and long neurites^23^.

### Quantitative assessment of motor neuron survival in the co-culture model

Motor neuron survival was evaluated as previously described^65^. Briefly, cells were labeled with an anti-SMI32 antibody to highlight motor neurons with typical morphology and large cell bodies (diameter ≥ 20 μm) and anti-NeuN antibody to identify all neurons. Twenty adjacent 10X-magnification frames per well were obtained using an Olympus IX71 microscope (Olympus) and analyzed using ImageJ Software. The ratio of the number of motor neurons to the total neurons was then calculated for each co-culture.

### Sample preparation for metabolomic analysis

NTG (AST^NTG^NEU^NTG^) and SOD1^G93A^ (AST^G93A^NEU^G93A^) co-cultures were obtained from non-transgenic (NTG) and SOD1^G93A^ (G93A) neurons seeded on NTG and SOD1^G93A^ astrocytes, respectively. Genotype mixed co-cultures were obtained using astrocytes and spinal neurons with different genotypes (AST^G93A^NEU^NTG^ and AST^NTG^NEU^G93A^). The co-cultures were grown for 3 days (3 DIV) or 6 days (6 DIV) after the seeding of spinal neurons on the astrocytes layer, without changing the medium.

Media from the co-cultures and from astrocytes in single culture were collected at 3 DIV or 6 DIV. For any co-culture, each individual sample (750 µl) was obtained making a pool of the medium from three wells. Media were transferred into a sterilised tube in microbiological safety cabinet and centrifuged (4 °C, 1000 rpm, 5 min) to pellet dead cells. Aliquots of the corresponding fresh (baseline, unexposed to cells) media were also collected. Then, supernatants were transferred to fresh tubes and stored at −80 °C until metabolomic analysis.

### ^1^H NMR spectroscopy of cell culture media

550 μL of culture medium and 50 μL of 11.6 mM 4,4-dimethyl-4-silapentane-1-ammonium trifluoroacetate (DSA) in deuterium oxide (D_2_O) as internal standard were mixed and transferred to glass 5 mm NMR tubes for analysis. High-resolution ^1^H NMR spectra of cell culture media were acquired at 14.1 T (600.13 MHz ^1^H frequency) using a Bruker AVANCE 600 spectrometer (Bruker, Rheinstetten, Germany). All spectra were acquired using a 1D presat Carr-Purcell-Meiboom-Gill (CPMG) spin-echo pulse sequence [RD-90°-(τ-180°-τ)n-AQ] with τ = 300 μs and *n* = 128.4 dummy scans and 64 transients were collected into 72 k data-points with a spectral width of 12019.230 and 0.3 Hz line broadening.

### Metabolomic data analysis

^1^H NMR spectra were imported and manipulated in Matlab (Mathworks, Natick, MA, USA) using in-house code for automatic phasing, baseline correction, and referencing chemical shifts to the DSA resonance at δ 0. The observed resonances were assigned to specific metabolites by database matching using the Chenomx NMR suite (Chenomx Inc., Alberta, Canada) and the Human Metabolome Database (HMDB). We calculated net consumption or net release of metabolites over the two sampling periods by subtracting metabolite concentrations in fresh/baseline medium from metabolite concentrations in spent medium from cultured cells (expressed as ΔRelative Concentration from Fresh Medium).

### Statistical analysis

The statistical significance of the data was tested by a two-way ANOVA followed by Tukey’s post-hoc test or by a Student’s t-test. All data are presented as mean ± SEM. Statistical analyses were performed in the R statistical environment.

## Electronic supplementary material

Supplementary information
