# Supplementary material for: Altered Metabolic Profiles Associate with Toxicity in SOD1G93A Astrocyte-Neuron Co-Cultures
Source: Sci Rep. 2017 Mar 3;7:50. doi: 10.1038/s41598-017-00072-4 (PMC5428359; doi:10.1038/s41598-017-00072-4)
Supplement: Supplementary file 1 — Supplementary information [file 41598_2017_72_MOESM1_ESM.pdf]

SUPPLEMENTARY INFORMATION

ALTERED METABOLIC PROFILES ASSOCIATE WITH TOXICITY IN SOD1^{G93A} ASTROCYTE-NEURON CO-CULTURES

Gabriel N Valbuena[1], Massimo Tortarolo[2], Caterina Bendotti[2], Lavinia Cantoni[3], and Hector C Keun[1]

[1]Department of Surgery and Cancer, Faculty of Medicine, Imperial College London, Du Cane Road, London W12 0NN

[2]Department of Neuroscience, and [3]Department of Molecular Biochemistry and Pharmacology, IRCCS- Istituto di Ricerche Farmacologiche "Mario Negri", 20156 Milan, Italy

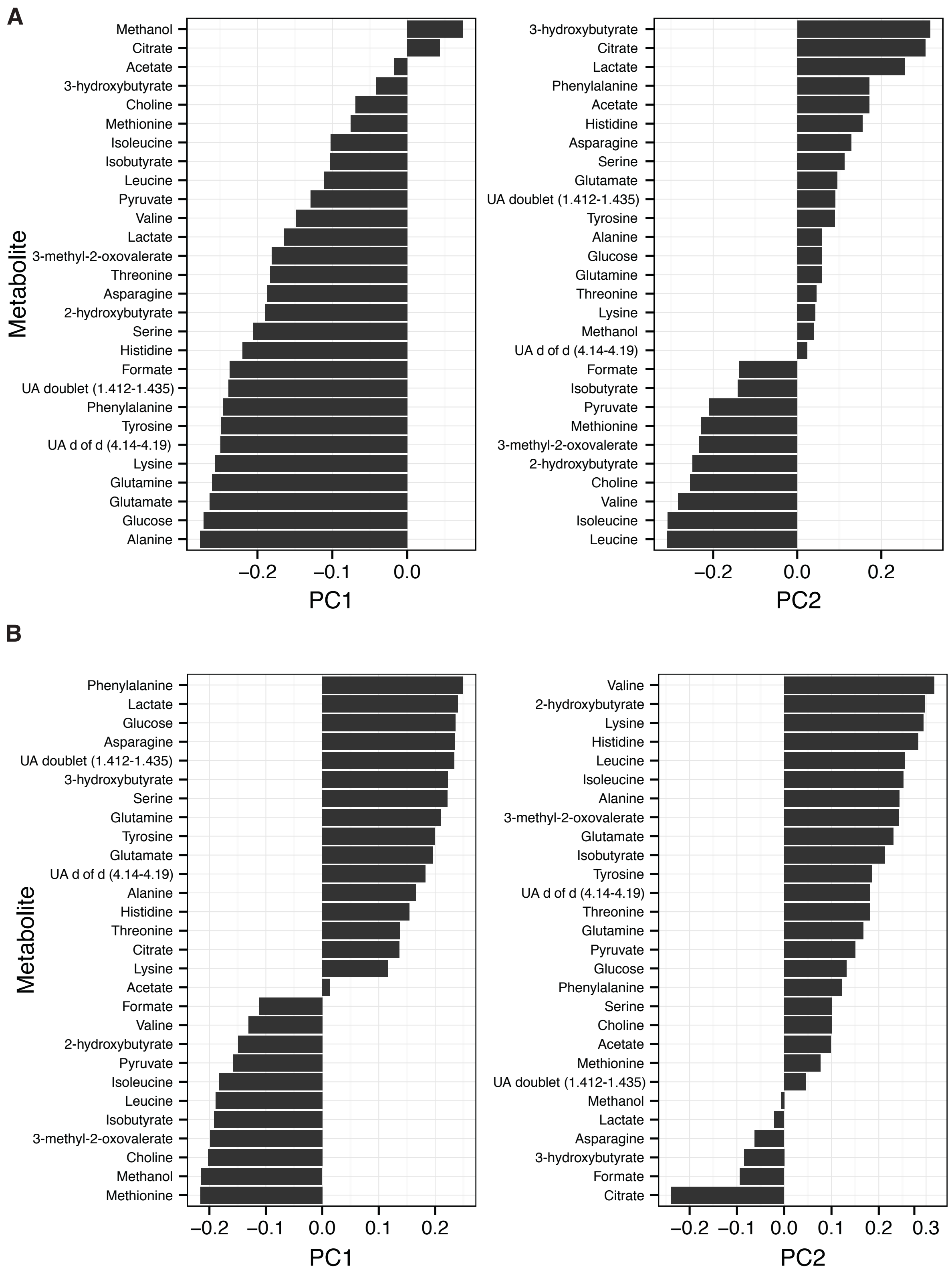

Supplementary Figure 1. PCA loadings for PC1 and PC2 from analysis of co-culture metabolomes after 3 and 6 days *in vitro*.

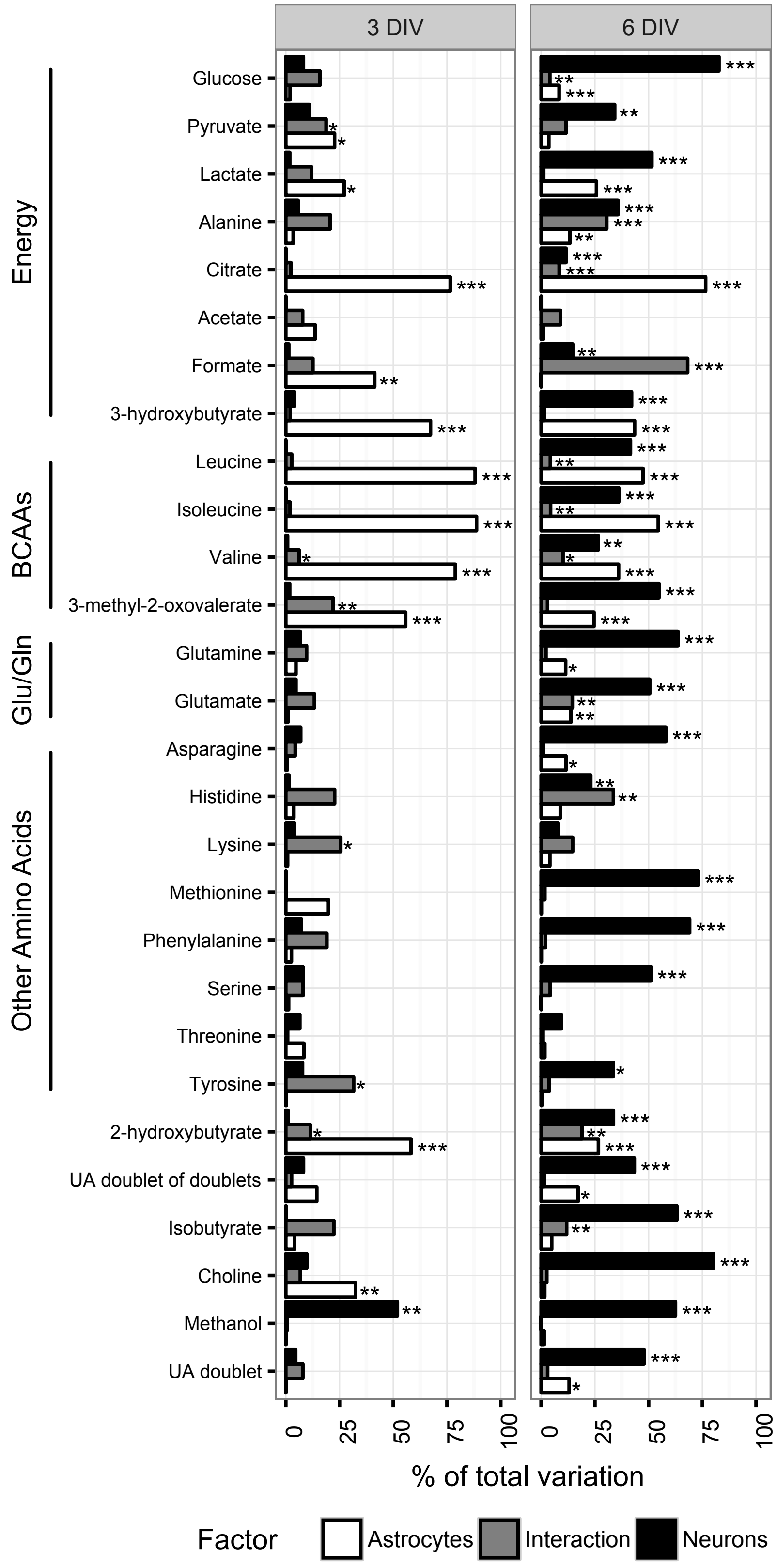

Supplementary Figure 2. Percentage contribution of astrocytes genotype, neuron genotype, and the interaction between the two to total variation in metabolite uptake and release in the astrocyte-neuron co-cultures. Percentage contribution and statistical significance shown were determined from a two-way ANOVA (*$p<0.05$, **$p<0.01$, *** $p<0.001$).

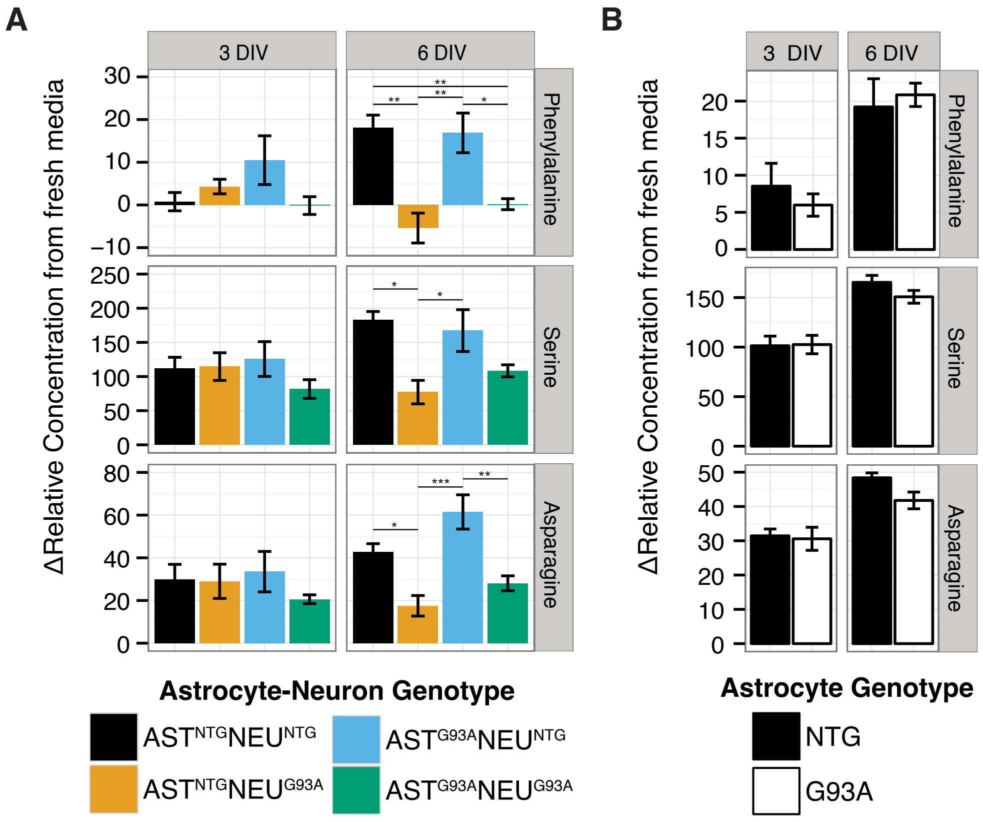

Supplementary Figure 3. Uptake and release of phenylalanine, serine, and asparagine from fresh media in (A) astrocyte-neuron co-cultures and (B) astrocyte single cultures. Asterisks denote statistically significant differences between groups after a one-way ANOVA followed by a Tukey HSD post hoc test for co-cultures and after a Student's t-test for astrocyte single cultures (*p<0.05, **p<0.01, *** p<0.001). All figures show mean ± s.e.m. (n = 4-5)

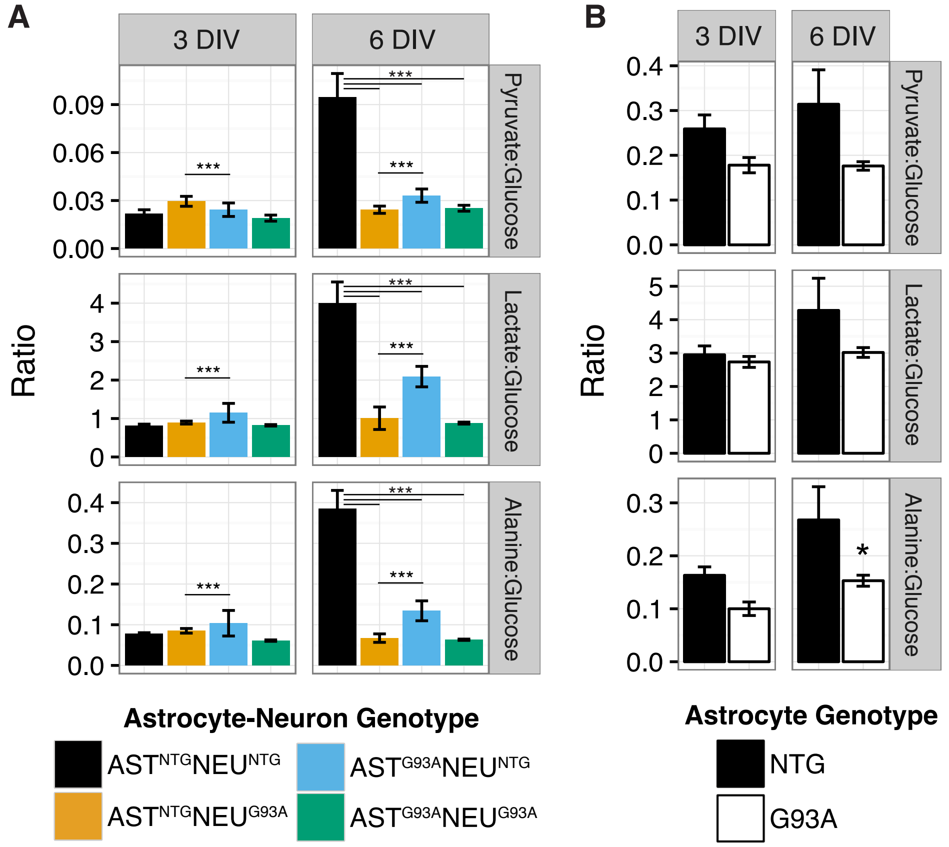

Supplementary Figure 4. Ratios of the release of pyruvate, lactate, and alanine to glucose uptake in media from (A) astrocyte-neuron co-cultures and (B) astrocyte single cultures. Asterisks denote statistically significant differences between groups after a one-way ANOVA followed by a Tukey HSD post hoc test for co-cultures and after a Student's t-test for astrocyte single cultures (*p<0.05, **p<0.01, *** p<0.001). All figures show mean ± s.e.m. (n = 4-5)

Table 1. Table of NMR spectral regions integrated

Metabolite	ppm region integrated	
	Lower limit	Upper limit
DSA (standard)	-0.02	0.02
2-hydroxybutyrate	0.88	0.9
Leucine	0.945	0.966
Isoleucine	0.9875	1.013
Valine	1.013	1.046
3-hydroxybutyrate	1.05	1.073
Isobutyrate	1.201	1.221
UA doublet	1.4125	1.435
Alanine	1.455	1.48
Acetate	1.901	1.914
Glutamate	1.985	2.05
Pyruvate	2.358	2.368
Glutamine	2.368	2.465
Methionine	2.61	2.655
Citrate	2.665	2.685
Lysine	3	3.04
Choline	3.18	3.195
Methanol	3.345	3.36
Serine	3.91	3.992
Asparagine	3.992	4.035
Lactate	4.075	4.14
UA doublet of doublets	4.14	4.19
Threonine	4.19	4.27
Glucose	5.21	5.245
Tyrosine	6.86	6.91
t-methylhistidine	7.02	7.055
Phenylalanine	7.3	7.34
Formate	8.442	8.45
